# Naringin Dihydrochalcone Ameliorates Cognitive Deficits and Neuropathology in APP/PS1 Transgenic Mice

**DOI:** 10.3389/fnagi.2018.00169

**Published:** 2018-06-05

**Authors:** Wenjuan Yang, Keyan Zhou, Yue Zhou, Yuqian An, Tingting Hu, Jing Lu, Shichao Huang, Gang Pei

**Affiliations:** ^1^State Key Laboratory of Cell Biology, CAS Center for Excellence in Molecular Cell Science, Shanghai Institute of Biochemistry and Cell Biology, Chinese Academy of Sciences, University of Chinese Academy of Sciences, Shanghai, China; ^2^School of Life Science and Technology, ShanghaiTech University, Shanghai, China; ^3^Shanghai Key Laboratory of Signaling and Disease Research, Laboratory of Receptor-based Bio-medicine, School of Life Sciences and Technology, Tongji University, Shanghai, China; ^4^School of Life Science and Technology, The Collaborative Innovation Center for Brain Science, Tongji University, Shanghai, China

**Keywords:** naringin dihydrochalcone, Alzheimer’s disease, amyloid-β, neuroinflammation, neurogenesis

## Abstract

Alzheimer’s disease (AD) is a multi-factorial neurodegenerative disorder with abnormal accumulation of amyloid-β (Aβ) plaques, neuroinflammation and impaired neurogenesis. Mounting evidences suggest that single-target drugs have limited effects on clinical treatment and alternative or multiple targets are required. In recent decades, natural compounds and their derivatives have gained increasing attention in AD drug discovery due to their inherently enormous chemical and structural diversity. In this study, we demonstrated that naringin dihydrochalcone (NDC), a widely used dietary sweetener with strong antioxidant activity, improved the cognitive function of transgenic AD mice. Pathologically, NDC attenuated Aβ deposition in AD mouse brain. Furthermore, NDC reduced periplaque activated microglia and astrocytes, indicating the inhibition of neuroinflammation. It also enhanced neurogenesis as investigated by BrdU/NeuN double labeling. Additionally, the inhibition of Aβ level and neuroinflammation by NDC treatment was also observed in an AD cell model or a microglia cell line. Taken together, our study indicated that NDC might be a potential therapeutic agent for the treatment of AD against multiple targets that include Aβ pathology, neuroinflammation and neurogenesis.

## Introduction

Alzheimer’s disease (AD) is the most common type of dementia and has no effective cure so far. Multiple factors are involved in the pathogenesis of AD: (1) the accumulation of amyloid-β (Aβ) and excessive Aβ (especially Aβ_42_) aggregation into plaques are considered the trigger of pathological events for AD (Holtzman et al., 2011); (2) neurofibrillary tangles composed of hyperphosphorylated tau protein contribute to neuronal dysfunction and involve in the progression of AD (Holtzman et al., 2011); (3) neuroinflammation is mediated by microglia and astrocytes and in response to brain damage (e.g., Aβ accumulation, neurofibrillary tangles), proinflammatory cytokines and mediators are produced, leading to chronic inflammation and neurodegeneration. (Bronzuoli et al., 2016); and (4) neurogenesis is reduced dramatically in AD, which can contribute to cognition impairment (Donovan et al., 2006; Drapeau and Nora Abrous, 2008). Multi-target compounds are proposed to tackle these factors and have attracted wide attention in recent years. For instance, curcumin and its derivatives which are suggested to have therapeutic potential for AD by inhibiting Aβ production and tau phosphorylation (Yang et al., 2005; Necula et al., 2007; Ma et al., 2009), stimulating embryonic neural stem cell proliferation via the MAP kinase pathways, and enhancing adult hippocampal neurogenesis (Kim et al., 2008). They are also reported to suppress inflammation process by reducing nuclear factor kappa-light-chain-enhancer of activated B cells (NF-κB)-mediated expression of proinflammatory cytokines (Jobin et al., 1999). These studies suggested that multi-target compounds might be promising drug candidates for AD.

Natural compounds and their derivatives have gained increasing attention due to their inherently enormous chemical diversity and many of them have turned into drug candidates (Lahlou, 2013; Guzior et al., 2015). Naringin is a major flavanone glycoside from Pomelo peel, and it has been experimentally demonstrated to improve long-term memory in the transgenic AD mouse model (Wang et al., 2013), it is interesting that whether its derivatives or other similar compounds may also have comparable or even better therapeutic effects on AD. Naringin dihydrochalcone (NDC) is a widely used natural compound derivative in food, medicine, and cosmetic industry as an artificial sweetener with antioxidant activity (Nakamura et al., 2003; Surana et al., 2006; Gaudette and Pickering, 2013). Large amount of evidence suggests that oxidative stress (OS) is involved in the development of AD (Feng and Wang, 2012), indicating NDC may have beneficial effects on the treatment of aging and neurodegenerative diseases. In our study, we investigated the effects of NDC on cognitive impairment and neuropathology in an AD mouse model. And the results suggest that oral administration of NDC ameliorated cognitive deficits, alleviated amyloid plaque burden and Aβ levels, suppressed neuroinflammation, and enhanced neurogenesis. Thus, NDC may be a promising multi-target drug candidate for the treatment of AD.

## Materials and Methods

### Ethics Statement

All animal experiments in this study were performed properly according to the Guide for the Care and Use of Laboratory Animals from National Institutes of Health. The protocols for animals were approved by the Research Ethics Committee, Shanghai Institutes for biological Sciences, Chinese Academy of Sciences. Animal pain and discomfort were minimized with enough food and water and other efforts.

### Animals and Drug Treatment

The APPswe/PS1ΔE9 (APP/PS1) transgenic mice (JAX Stock No. 004462) expressing mouse/human amyloid precursor protein (Mo/HuAPP695swe) and human Presenilin1 (PS1ΔE9) were used in our investigation and mice were maintained and genotyped according to the guidelines of Jackson Laboratory. The wild type (WT) littermates were used as age- and gender-matched controls. NDC (with a purity 95%–99%; Biopurify Phytochemicals Ltd., Chengdu, China^1^) was dissolved in vehicle (H_2_O). WT and APP/PS1 mice chronically administered 200 μl of NDC (100 mg/kg) or vehicle per 20 g mouse body weight body weight by oral administration once a day from 3–4 to 6–7 months of age (*n* = 10–12 mice per group).

For assessment the effect of NDC on neurogenesis, these mice were intraperitoneally injected with 5-bromo-2-deoxyuridine (BrdU, Sigma, 50 mg/kg/d) once a day, from days 73 to 79 during drug administration, as described by Encinas et al. (2011).

### Morris Water Maze

The Morris Water Maze (MWM) were carried out as previously described (Morris, 1984; Teng et al., 2010). The apparatus was a 120-cm-diameter circular water pool containing small white plastic particles, with four different cues located on the four directions of pool wall. At a fixed position in the target quadrant, 11-cm- diameter transparent platform was placed 1 cm below the water surface. During the whole experiment, the water temperature was maintained at 21.0 ± 0.5° and the room temperature was 23.0 ± 0.5°. The training consisted of four trials 1 day for six consecutive days. To assess spatial memory, on day 7, we performed a probe trial. During the probe trial, the platform was removed away from the target quadrant of water pool and mice were allowed to explore for 1 min freely. All tracks from all trials were monitored using an automated tracking system (Ethovision XT software) for the animal performance analysis.

### Novel Object Recognition

The Novel Object Recognition test (NOR) is also widely used to evaluate recognition memory in mice. The detailed protocol with modifications is as previously described (Bevins and Besheer, 2006; Liu et al., 2013; Hou et al., 2014). The procedure included two phases: training phase and testing phase. On the first day, mice were placed in an evenly illuminated sound proof box with a Plexiglas box (25 cm × 25 cm × 25 cm). In the presence of two equal objects, each mouse was allowed to explore for 10 min freely. On the second day, one of the equal objects was replaced by a novel, unfamiliar object. Animals were placed back in the arena to freely explore for 10 min. During the whole trial, to eliminate olfactory cues, the arena and objects were cleaned thoroughly with 10% ethanol. Object exploration time was the time of a mouse was sniffing, directing and pawing the object. The time was recorded in a double-blinded manner. In the training phase, location preference means the time of a mouse exploring one object relative to the time of exploring two objects, and in the testing phase, recognition index means the time of a mouse exploring the novel object relative to the time of exploring two objects.

### ELISA for Human Aβ and Western Blot Analysis

Aβ_40_ and Aβ_42_ in APP/PS1 mouse hippocampus and cortex were extracted as previously reported (Lazarov et al., 2005), for ELISA measurement of human Aβ_40_ and Aβ_42_, the frozen hippocampal and cortical tissue (400 mg) stored in −80°C were homogenized in 1 ml 2% SDS (dissolved in PBS), then centrifuged at 1,20,000 *g* for 60 min at room temperature. The supernatant was collected as the soluble fraction and quantified with human Aβ ELISA kits according to the users’ guidelines (ExCell Bio). Total Aβ levels in HEK293/APPswe cell culture medium were also quantified with ELISA.

Supernatant is also used for Western blot analyses. Proteins in supernatant were separated by SDS-PAGE, and transferred onto membrane. Proteins were labeled with β-actin (AB0035, Abways) and IL-1β rabbit polyclonal antibody (16806-1-AP, Proteintech) and the immunoreactive bands were detected by chemiluminescent detection (Bio-Rad) of peroxidase-conjugated antibody (M21002, Abmart). The intensity of each band was quantified by ImageJ and normalized to β-actin.

### Immunohistochemistry and Image Analysis

After behavioral tests, the mice were anesthetized with chloral hydrate and transcardially perfused with phosphate-buffered saline (PBS) buffer and then with 4% paraformaldehyde (PFA) in PBS. The half brain tissue were serially sectioned at 30 μm thickness and stained using Thioflavin S (ThioS; Sigma) and anti-Aβ antibody 6E10 (Covance, SIG-39300) for amyloid plaques, a polyclonal rabbit antibody against GFAP (DAKO) for astrocytes, a mouse antibody against Iba1(WAKO) for microglia and anti-NeuN antibody for neurons.

According to previous studies (Galea et al., 2015; Krauthausen et al., 2015), GFAP-positive astrocytes within an 80 μm radius surrounding Aβ plaques were quantified in hippocampus and cortex. The proportion of GFAP-positive areas in cortex means the GFAP-positive areas in cortex relative to total areas of cortex, and the same in hippocampus.

All images of brain slices were captured using a confocal laser scanning microscope (Leica TCS SP8). Each section was captured by confocal microscopy in z stack, covered all layers of cells and all positive-staining cells were counted. For counting the NeuN^+^BrdU^+^ double-stained cells, the co-localization of different channels in each cell was carefully confirmed. Quantification was performed using ImageJ software and the percentage of antibody-positive area was calculated. Five to six sections were analyzed per mouse and all assessments were analyzed in a blinded manner.

### Cell Culture and Treatment

HEK293 cells were purchased from ATCC. HEK293/APPswe cells were transfected, selected and maintained in our lab. BV2 cell lines were cultured and maintained in Dulbecco’s Minimal Essential Medium (DMEM), with 10% fetal bovine serum and 100 U/ml penicillin and 0.1 mg/ml streptomycin. HEK293/APPswe cells were cultured in MEM under the same condition. BV2 cells were treated with 0.3 μg/ml lipopolysaccharides (LPS; 055:B5, Sigma) and various concentrations of NDC for 6 h. HEK293/APPswe cells were treated with various concentrations of NDC for 24 h.

### Quantitative Real-Time PCR

The analysis of mRNA expression was performed as previously reported (Cai et al., 2017), 3e4 BV2 cells per well were seeded into 96-well plates. After NDC treatment, total RNA was extract according to the instructions of TRI Reagent^®^ (Sigma) and used NanoDrop 1000 Spectrophotometer (Thermo Scientific) to assess the RNA purity and integrity. TIANScript M-MLV kit (TIANGEN) was used to synthesis cDNA according to protocols, and rRNasin^®^ (Recombinant rRNasin^®^ Ribonuclease Inhibitor, Promega) was used in the synthesis. The expression level of mRNA was measured by quantitative Real-time PCR (qPCR) using the 2× HotStart SYBR Green qRT-PCR Master Mix kit from ExCell. The reaction parameters were: 95°C for 10 min; 95°C for 30 s, 40 cycles; 60°C for 30 s; 72°C for 30 s. An additional cycle was performed for evaluation of primer’s dissociation curve: 95°C for 1 min, 60°C for 30 s and 95°C for 30 s. Each cDNA sample was amplified in duplicates. Primer sequences are listed in Table 1.

**Table 1:** Primers used for qPCR.

Gene	Direction	Sequence
Il-1β	F	GTTGACGGACCCCAAAAGAT
	R	AAGGTCCACGGGAAAGACAC
Il-6	F	TAGTCCTTCCTACCCCAATTTCC
	R	TTGGTCCTTAGCCACTCCTTC
TGF-β	F	CACTGATACGCCTGAGTG
	R	GTGAGCGCTGAATCGAAA
TNF-α	F	ACCCTCACACTCAGATCATCTTC
	R	TGGTGGTTTGCTACGACGT

### CellTiter-Glo Assay

Cell viability of NDC-treated HEK293/APPswe or BV2 cells was investigated with CellTiter-Glo luminescent Cell Viability Assay (Promega) according to the manufacturer’s instructions.

### Statistical Analysis

All data are presented as mean ± SEM. Statistical analysis was performed with GraphPad Prism 6 Software (San Diego, CA, USA). Results were analyzed by two-tailed *t*-test to determine the statistical significance of treatment sets. For multiple comparisons, one-way ANOVA or two-way ANOVA were performed, followed by Bonferroni test. Differences were considered significant when *p* < 0.05.

## Results

### NDC Ameliorates Learning and Memory Deficits in APP/PS1 Mice

**Figure 1:**
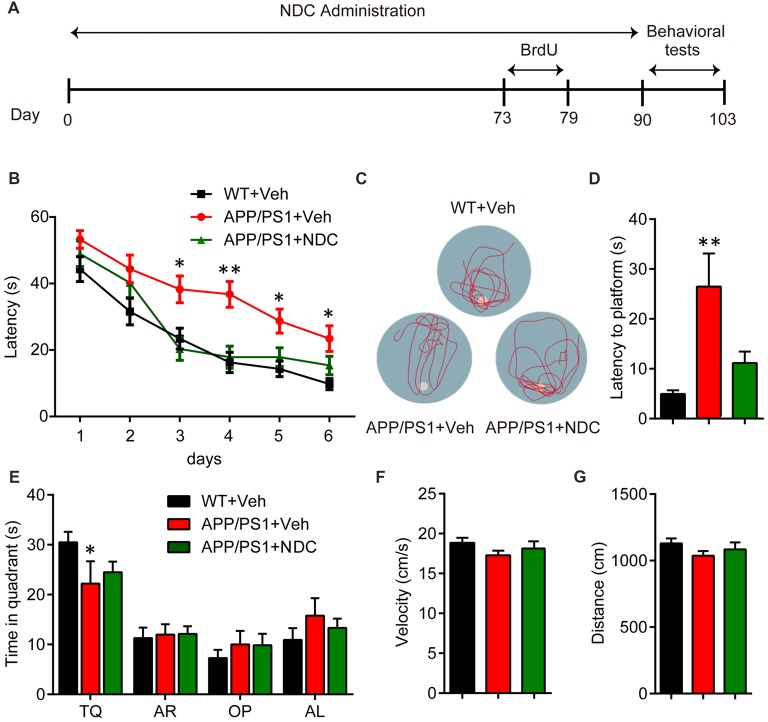
Naringin dihydrochalcone (NDC) ameliorates learning and memory deficits in APP/PS1 mice. **(A)** Experimental procedure for Morris water maze (MWM) analysis. **(B)** MWM test of wild type (WT) mice and vehicle- or NDC-treated APP/PS1 mice. **(C)** Representative track images of each group mice in day 7 probe trial test. **(D)** Latency to platform of each group mice in day 7 probe trial test. **(E)** Time spent of each group mice in the target quadrant in day 7 probe trial test. TQ, target quadrant; AR, adjacent right; OP, opposite; AL, adjacent left. **(F)** Swimming velocity of each group mice in probe trial. **(G)** Swimming distance of each group mice in probe trial. Data are presented as mean ± SEM, WT group *n* = 8, Veh group *n* = 7, NDC group *n* = 7, *p < 0.05, **p < 0.01, analyzed by two-way ANOVA **(B,E)** or one-way ANOVA **(D,F,G)** followed by Bonferroni test.

To investigate the potential therapeutic of NDC for AD, we began the oral administration of NDC from 3- to 4-month-old APP/PS1 mice for 3 months. During drug administration, all treated animals’ body weight was recorded and there was no body weight loss or obvious adverse effects among groups (data not shown). After drug administration, the behavioral tests were performed to investigate the cognitive function of these mice. MWM was carried out to assess spatial learning and memory ability (Figure 1A). The latency to platform was analyzed using two-way ANOVA (*F*_(2,114)_ = 18.65, *p* < 0.001). We found that compared with WT mice, APP/PS1 mice spent more time in locating the platform (*p* < 0.001), indicating it exhibited significant cognitive decline in learning, and there was no significant difference between NDC-treated mice and WT mice (*p* > 0.05), indicating that the cognitive function of spatial memory was significantly improved by treatment of NDC (Figure 1B). On day 7, probe trials were performed to assess the maintenance of spatial memory. Compared with WT mice, APP/PS1 vehicle mice crossed the platform position was less frequently (Figure 1C) and spent more time to reach position of missing platform. However, no significant difference in latency was observed between NDC-treated mice and WT mice (*F*_(2,19)_ = 8.650, *p* < 0.005; Figure 1D).The time in quadrant was Two-way ANOVA revealed no significant effect for group (*F*_(2,76)_ = 0.000097, *p* > 0.05), compared with WT mice, APP/PS1 mice spent less time in target quadrant (*p* = 0.0415; Figure 1E). Among three groups, there was no significant difference in velocity (*F*_(2, 19)_ = 1.340, *p* > 0.05) or distance (*F*_(2,19)_ = 1.334, *p* > 0.05) of swimming, suggesting that NDC treatment did not influence locomotor activity of mice (Figures 1F,G). These results suggest that administration of NDC ameliorates the spatial learning and memory of APP/PS1 mice.

**Figure 2:**
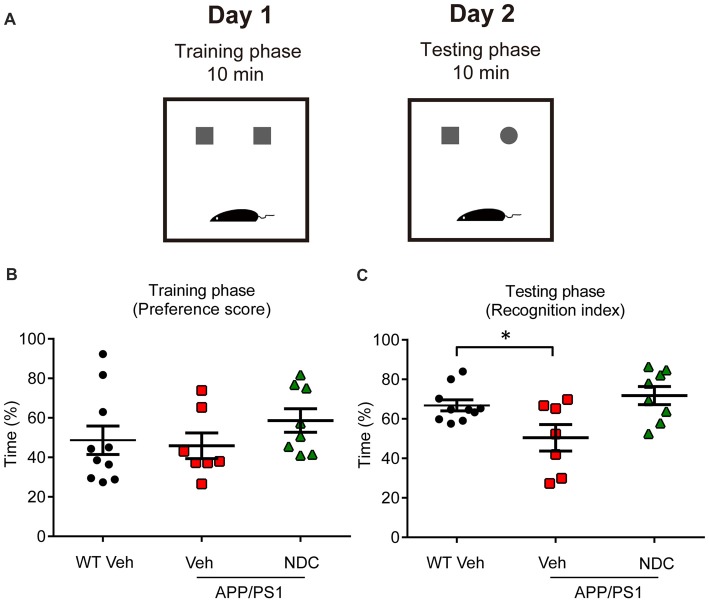
NDC improves memory retention in APP/PS1 mice. **(A)** Diagram of Novel object recognition (NOR) analysis. **(B,C)** Preference scores of training phase **(B)** and Recognition Index of testing phase **(C)** during a 10-min testing phase are shown, respectively. Data are presented as mean ± SEM, WT group *n* = 10, Veh group *n* = 7, NDC group *n* = 8. *p < 0.05, analyzed by one-way ANOVA followed by Bonferroni test.

To further evaluate the learning and recognition memory of AD mice, we performed the NOR test (Figure 2A). In the training phase, there was no significant difference among groups, the preference scores were all about 50% in all groups (Figure 2B). In the testing phase, APP/PS1 vehicle mice spent less time to explore the novel object than WT mice, NDC-treated APP/PS1 mice spent much longer time to explore the novel object than APP/PS1 vehicle mice (*F*_(2,22)_ = 5.412, *p* < 0.05), the recognition index of NDC-treated mice was similar with that of WT mice (Figure 2C), indicating that NDC-treatment efficiently improved memory retention of APP/PS1 mice.

### NDC Alleviates Amyloid Plaque Burden and Aβ Levels in APP/PS1 Mice

**Figure 3:**
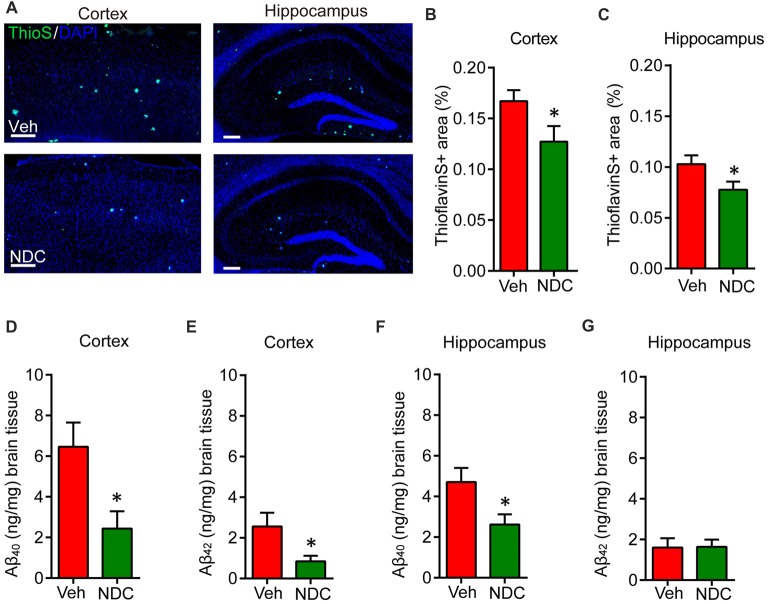
NDC alleviates amyloid plaque burden and Aβ levels in APP/PS1 mice. **(A)** Representative half brain sections of vehicle- or NDC-treated APP/PS1 mice brain stained with ThioS and DAPI in cortex and hippocampus are shown. Scale bar = 200 μm. **(B,C)** Quantitative analysis of the of ThioS-positive amyloid plaques covered area in cortex **(B)** and hippocampus **(C)**. **(D,E)** ELISA of soluble Aβ_40_ and Aβ_42_ levels in cortical tissues of APP/PS1 mice. **(F,G)** ELISA of soluble Aβ_40_ and Aβ_42_ levels in hippocampal tissues of APP/PS1 mice. Data are presented as mean ± SEM. ThioS: Veh group *n* = 5, NDC group *n* = 5; ELISA: Veh group *n* = 6, NDC group *n* = 6. *p < 0.05, analyzed by two-tailed *t* test compared with APP/PS1 vehicle group.

The Aβ levels or deposits in the brain of APP/PS1 mice can be detected on 6 month. To explore the effects of NDC on Aβ levels and Aβ deposits, ThioS staining for amyloid plaques was performed on these fixed brain tissues (Figure 3A). Detailed statistical analysis showed that, ThioS-positive amyloid plaque areas were markedly reduced in the brains of NDC-treated APP/PS1 mice compared with that in the brains of vehicle-treated APP/PS1 mice (Figures 3B,C), and 6E10-positive amyloid plaque areas were also significantly reduced (Supplementary Figure S1), indicating that NDC treatment alleviates the deposition of amyloid plaques.

Simultaneously, we performed ELISA assay to quantify Aβ levels in the cortex and hippocampus of these mice. Results showed that the Aβ levels were high in cortex and hippocampus of APP/PS1 transgenic mice. NDC treatment significantly reduced SDS-soluble Aβ_40_ and Aβ_42_ levels in cortex (Figures 3D,E). In hippocampus, the level of SDS-soluble Aβ_40_ was also reduced significantly (Figure 3F), but there is no significant differences between the hippocampus Aβ_42_ level in NDC-treated and vehicle-treated mice (Figure 3G). These results suggest that NDC reduced Aβ levels in APP/PS1 mice brain.

**Figure 4:**
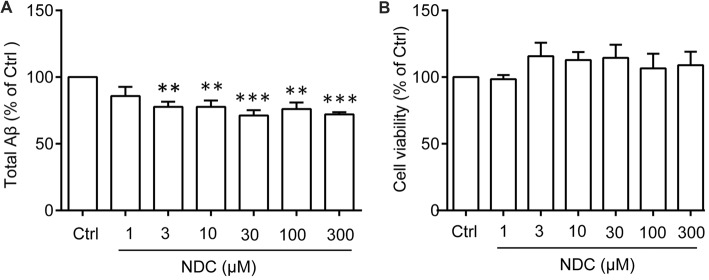
NDC reduces Aβ level in HEK293/APPswe cells. **(A)** The total Aβ level in HEK293/APPswe culture medium. **(B)** Cell viability of HEK293/APPswe cells after treatment with NDC for 24 h. Data are presented as mean ± SEM, *n* = 3 independent experiments, **p < 0.01, ***p < 0.005, analyzed by one-way ANOVA **(A,B)** followed by Bonferroni test.

To identify whether NDC could reduce Aβ level *in vitro*, we treated HEK293/APPswe cells with various concentrations of NDC. The culture medium was collected to evaluate total Aβ level using ELISA assay and the cell were used to evaluate cell viability. Compared to the vehicle treatment, NDC significantly reduced the total extracellular Aβ level (*F*_(6,28)_ = 5.459, *p* < 0.001; Figure 4A). The viability of cells was not declined after 24 h treatment as revealed by the CellTiter-Glo assay (*F*_(6,14)_ = 0.3613, *p* > 0.05; Figure 4B). These results suggest that NDC can reduce Aβ generation in an AD cell model.

### NDC Attenuates Neuroinflammation in APP/PS1 Mice

**Figure 5:**
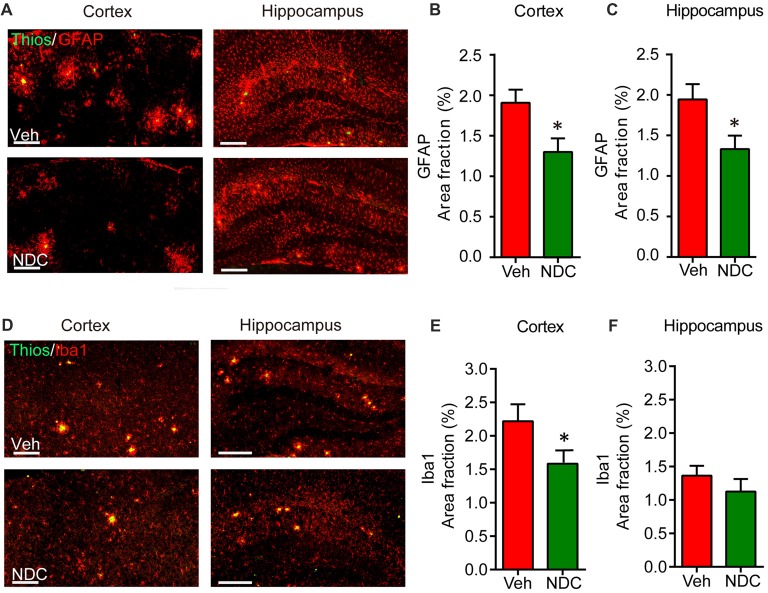
NDC reduces periplaque activated microglia and astrocytes in APP/PS1 mice. **(A)** Representative sections of vehicle- and NDC-treated APP/PS1 mice brain immunostained with ThioS and GFAP in cortex and hippocampus are shown. Scale bar = 200 μm. **(B,C)** Quantitative analysis of ThioS and GFAP double staining in cortex **(B)** and hippocampus **(C)**. **(D)** Representative sections of vehicle- and NDC-treated APP/PS1 mice brain immunostained with ThioS and microglial makers Iba1 in cortex and hippocampus are shown. Scale bar = 200 μm. **(E,F)** Quantitative analysis of ThioS and Iba1 double staining in cortex **(E)** and hippocampus. **(F)** Data are presented as mean ± SEM, *n* = 5 per group, *p < 0.05, analyzed by two-tailed *t*-test compared with APP/PS1 vehicle group.

Abnormal neuroinflammation, including activated astrocytes and microglia, is a typical hallmark of neurodegenerative disease, and amyloid plaques are surrounded by activated microglia and astrocytes in the brains of AD patients (McGeer and McGeer, 2003). To investigate its possible anti-inflammatory effects of NDC in APP/PS1 mice, we stained brain sections with polyclonal antibodies against Iba1 and GFAP, which are indications of active microglia and astrocytes, respectively. As previously reported (Hou et al., 2014), we observed that amyloid plaques were surrounded by GFAP-positive astrocytes and Iba1-positive microglia. Compared with vehicle-treated APP/PS1 mice, we found that NDC-treated APP/PS1 mice showed lower GFAP-positive area and Iba1-positive area in cortex and hippocampus (Figures 5A,D). Further statistical analysis demonstrated that compared with the vehicle-treated APP/PS1 mice, the proportion of GFAP-positive areas surrounding Aβ in the cortex and hippocampus of NDC-treated APP/PS1 mice were reduced (Figures 5B,C). Similarly, we found the proportion of Iba1-positive microglia areas surrounding Aβ also decreased in the cortex and hippocampus (Figures 5E,F). These results demonstrate that NDC treatment could significantly reduce the area fraction of activated microglia and astrocytes in APP/PS1 Mice.

**Figure 6:**
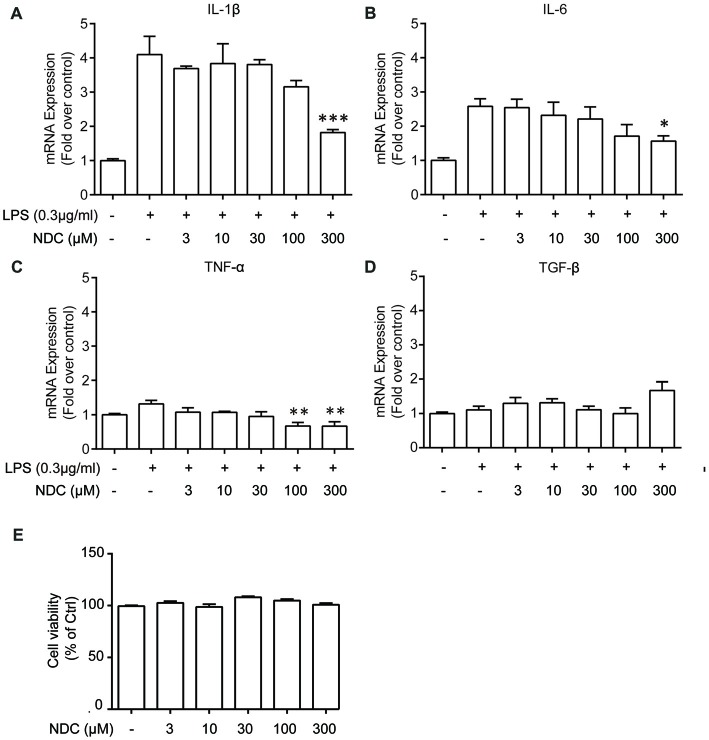
NDC reduces levels of inflammation cytokines in BV2 cells. **(A–D)** Quantitative PCR (qPCR) quantification of proinflammatory cytokines IL-1β **(A)**, TNF-α **(B)**, IL-6 **(C)** and one anti-inflammatory cytokine, TGF-β **(D)**. **(E)** Cell viability of BV2 cells after 6 h NDC treatment. Data are presented as mean ± SEM, *n* = 3 independent experiments, *p < 0.05, **p < 0.01, ***p < 0.005, analyzed by one-way ANOVA followed by Bonferroni test revealed difference between NDC- and lipopolysaccharides (LPS)-treated condition **(A–D)**, as well as difference between control and NDC-treated condition **(E)**.

We then investigated the effect of NDC on the LPS-induced microglia activation. The BV2 cells were incubated with various concentrations of NDC in the presence of LPS (0.3 ug/mL) for 6 h. qPCR results demonstrated that NDC inhibited LPS-induced proinflammatory cytokines, interleukin 1 beta (IL-1β; *F*_(6,24)_ = 13.04, *p* < 0.001), Interleukin 6 (IL-6; *F*_(6,32)_ = 6.350, *p* < 0.001), and tumor necrosis factor alpha (TNF-α) expression (*F*_(6,23)_ = 4.825, *p* < 0.01; Figures 6A–C). These cytokines were effectively reduced mainly by NDC at 300 μM. In addition, NDC slightly up-regulated the expression of anti-inflammatory cytokine transforming growth factor beta (TGF-β) in the presence of LPS (Figure 6D), and NDC had no obvious cytotoxicity to BV2 cells as assessed by the CellTiter-Glo assay (Figure 6E). Furthermore, Western blot results suggested that NDC also reduced IL-1β level in AD mouse brain (Supplementary Figure S2). Thus, our results indicate that NDC inhibited the expression of proinflammatory cytokines and promoted the expression of anti-inflammatory cytokines.

### NDC Enhances Neurogenesis in APP/PS1 Mice

**Figure 7:**
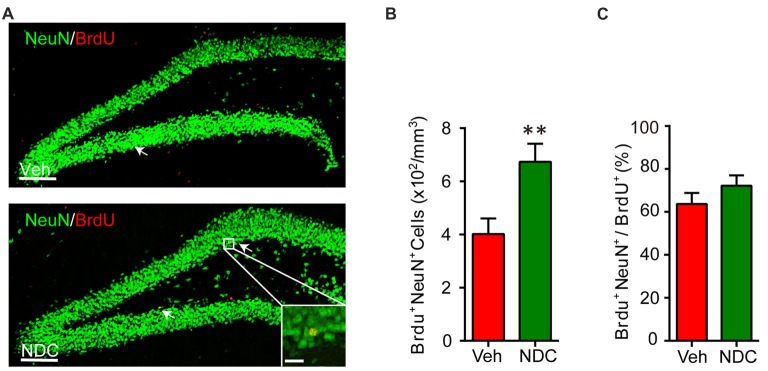
NDC enhances neurogenesis in APP/PS1 mice. **(A)** NeuN (green) and BrdU (red) staining in dentate gyrus (DG) of brain sections from mice treated with vehicle **(A)** and NDC **(B)**. **(B)** Quantification of BrdU^+^ NeuN^+^ cells in the brain sections of vehicle- and NDC-treated mice. **(C)** Proportion of BrdU^+^ NeuN^+^ cells in all BrdU^+^ cells. Data are presented as mean ± SEM, Veh group *n* = 7, NDC group: *n* = 8, **p < 0.01, analyzed by two-tailed *t*-test **(B,C)** compared with APP/PS1 vehicle group. Scale bars in **(A)**, 100 μm; insets, 20 μm.

In AD patients and animal models, abnormal neurogenesis is related to cognitive decline. We investigated whether NDC could affect neurogenesis in APP/PS1 mice with the improvement in cognition. APP/PS1 mice were intraperitoneally injected with bromodeoxyuridine (BrdU) for 7 days and then euthanized after behavioral tests. Brain sections were stained with BrdU/NeuN (Figure 7A). Compared with vehicle-treated APP/PS1 mice, the number of BrdU/NeuN double-positive cells in the hippocampus of NDC-treatment APP/PS1 mice was increased significantly (Figure 7B). However, the proportion of BrdU/NeuN double-positive cells in the BrdU-positive cells was not altered among two groups after NDC treatment (Figure 7C). These results indicated that NDC treatment promotes neurogenesis in APP/PS1 transgenic mice.

## Discussion

In this study, NDC significantly ameliorated cognitive impairment and neuropathology in transgenic AD mice through reducing amyloid plaque burden and Aβ levels, suppressing neuroinflammation and promoting neurogenesis, which is in line with previous findings on other natural compounds, such as curcumin and Naoling decoction (Ono et al., 2002; Hamaguchi et al., 2006, 2010; Hatcher et al., 2008; Xia et al., 2017). Taken together, these studies suggest that natural compounds such as NDC could achieve multi-target directed therapy in the treatment of AD.

In current AD drug research, many targets or strategies are considered, such as preventing Aβ accumulation or tau phosphorylation, inhibition of secretase activity that directly modulates Aβ generation, OS, neuroinflammation, as well as mitochondrial damage (Tundis et al., 2018). On the basis of these targets and processes, most therapeutic strategies for AD focus on Aβ and phosphorylated tau levels and OS (Selkoe, 2001; Dysken et al., 2014a). However, it has been observed that drugs directed to a single target with high specificity often lacked clinical efficacy on AD, highlighting the complexity of this disorder (León et al., 2013). Notably, antioxidants such as rutin, resveratrol and vitamin E have been reported to exert beneficial effects on the treatment of AD (Zuo et al., 2015). Interestingly, in addition to its antioxidant effect, vitamin E can also reduce Aβ levels and amyloid deposition in the brain of transgenic AD mice (Sung et al., 2004). It also suppresses inflammatory responses (reduction of GFAP, IL-1β) and decreases tau pathology (Nakashima et al., 2004; Yao et al., 2004). Clinical trials further showed that vitamin E has protective effects in patients with mild to moderate AD and the cognitive performance of patients was changed significantly (Dysken et al., 2014b). In our study, NDC exhibited beneficial effects on Aβ, NDC reduced Aβ levels and alleviated the deposition of amyloid plaques in AD mice similar with vitamin E. Neurogenesis is an essential process to maintain hippocampus-dependent cognitive abilities (Deng et al., 2010), and the impaired neurogenesis is involved in the progression of AD (Martinez-Canabal, 2014). Here, NDC also enhanced neurogenesis in transgenic mice model. All these suggest that the beneficial effects of NDC may not only due to its antioxidant activity but also possess other activities.

In previous studies, the relationship between neuroinflammation and Aβ is complicated. On the one hand cerebral inflammation could increase Aβ production (Iadecola, 2003; Walker and Lue, 2007), and on the other hand aggregated Aβ peptide could activate microglia to a proinflammatory state (Walker and Lue, 2005, 2007). In our study, NDC not only reduced the activated microglia and astrocytes but also reduced Aβ plaques in AD mice brain. It has been reported that NDC possesses anti-inflammatory properties as an antioxidant (Nakamura et al., 2003). Therefore we guess that in our study NDC may suppress the neuroinflammation, which further leads to the decrease of Aβ production. What’s more, we also found that NDC treatment reduced production of inflammatory cytokines in BV2 cells, which further suggests NDC may affect microglia directly. To prove this hypothesis, more *in vitro* assays including treatment of NDC and Aβ in primary microglia culture should be carried out.

In summary, the current study first demonstrates the protective effects of NDC on AD pathology by targeting multiple processes in an AD mouse model, which suggests that NDC may serve as a promising therapeutic agent for AD.

## Author Contributions

GP substantially designed and controlled the study. WY and YZ performed the behavioral tests. KZ, WY and YZ performed histopathological and data analysis. YA and TH performed the *in vitro* experiments. KZ and WY contributed to the manuscript preparation. SH and JL contributed to critical revision of the manuscript. All the authors reviewed and commented on the manuscript.

## Conflict of Interest Statement

The authors declare that the research was conducted in the absence of any commercial or financial relationships that could be construed as a potential conflict of interest.

## Footnotes

^1^http://www.biopurify.com
