# Supplementary material for: Naringin Dihydrochalcone Ameliorates Cognitive Deficits and Neuropathology in APP/PS1 Transgenic Mice
Source: Front Aging Neurosci. 2018 Jun 5;10:169. doi: 10.3389/fnagi.2018.00169 (PMC5996202; doi:10.3389/fnagi.2018.00169)
Supplement: Supplementary file 1 [file Data_Sheet_1.pdf]

Supplementary Material

Naringin Dihydrochalcone Ameliorates Cognitive Deficits and Neuropathology in APP/PS1 Transgenic Mice

Wenjuan Yang*, Keyan Zhou, Yue Zhou

Correspondence: Corresponding Author: Huangshichao@sibcb.ac.cn and gpei@sibs.ac.cn

Supplementary Figures

Figure S1. NDC alleviates 6E10-positive plaque in APP/PS1 mice. Related to Figure 3.

Figure S2. NDC reduces the level of IL-1β in APP/PS1 mice. Related to Figure 6

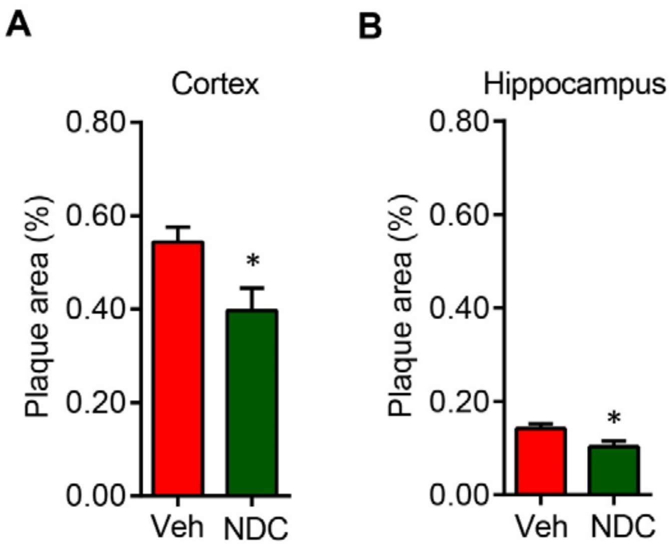

Figure S1. NDC alleviates 6E10-positive plaque in APP/PS1 mice.

(A and B) Quantitative analysis of the of 6E10-positive amyloid plaques covered area in cortex (A) and hippocampus (B).

Data are presented as mean ± SEM, n = 5 per group. **P* < 0.05, analyzed by two-tailed t test compared with APP/PS1 vehicle group,

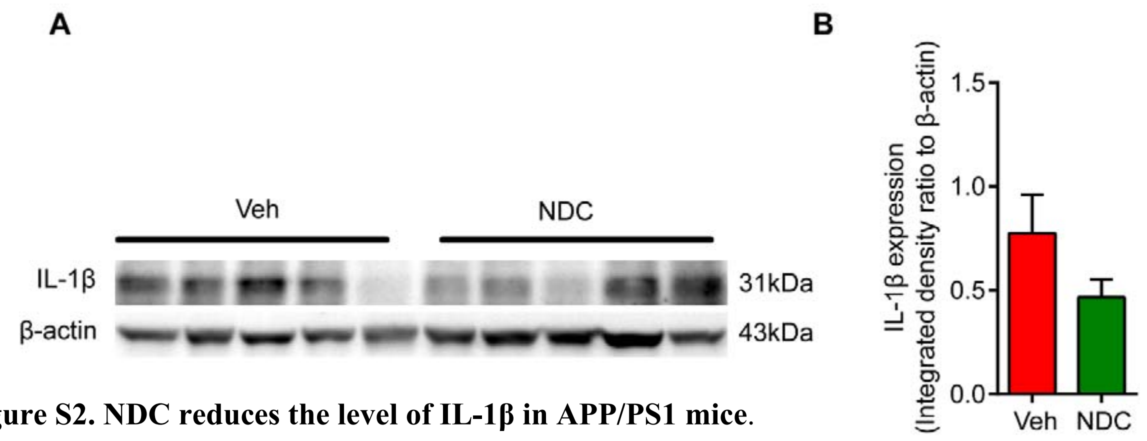

Figure S2. NDC reduces the level of IL-1β in APP/PS1 mice.

(A) Western blot analysis of IL-1β level.

(B) Quantitative analysis of the IL-1β level in APP/PS1 mice.

Data are presented as mean ± SEM, n = 5 per group. *P* =0.17, analyzed by two-tailed t test compared with APP/PS1 vehicle group,
